# *Plant Physiology* welcomes 19 new assistant features editors

**DOI:** 10.1093/plphys/kiac537

**Published:** 2022-12-02

**Authors:** Yunde Zhao, Mike Blatt, Mary Williams

**Affiliations:** Section of Cell and Developmental Biology, University of California San Diego, 9500 Gilman Drive, La Jolla, California 92093-0116, USA; Laboratory of Plant Physiology and Biophysics, University of Glasgow, Glasgow G12 8QQ, UK; American Society of Plant Biologists, USA

*Plant Physiology* initiated the Assistant Features Editor (AFE) program five years ago to help disseminate discoveries published in the journal and to train the next generation of editors and reviewers. Our AFEs are promising early-career scientists and they bring their passion for science to our journal, communicating to our readers each month some of the most exciting advances in research.

The AFEs have added substantially to the plant science community and to the journal. Their contributions have expanded our content through *News and Views* articles, blog posts, and related material highlighting content of special interest in *Plant Physiology*. They have grown professionally and will build on their experience with the journal. Many of our previous AFEs have moved onto independent academic positions after completing their terms at *Plant Physiology*. We hope to call the AFEs to serve as regular members of the editorial board when they have become more established in their careers.

The program is designed to rotate off a number of AFEs each year and to add new members at the beginning of each year. Sixteen of our AFEs stepped down from the editorial board this past month, and we would like to take this opportunity to thank them all for their contributions. We would also like to welcome the 19 new AFEs (listed below; pictures available https://plantae.org/2023-plant-physiology-assistant-features-editors/) who started last month and will work alongside our seasoned crew.

We, too, have learned much from working with the AFEs these past five years. We have honed the way we support them in writing *News and Views* articles and have streamlined oversight through the journal online submission system. We have invited them to write profiles of more senior members of our editorial board, which we publish on our Plantae blog. AFEs are invited to attend the annual editorial board meetings of *Plant Physiology* where they can learn the intricacies of editorial process and contribute to the discussions of *Plant Physiology* policies and operation. Additionally, in response to feedback from the AFEs, we are working to formalize aspects of their training and are building an AFE mentorship program.

So, welcome! We are thrilled to have both the new and returning AFEs with us. We want, also, to add our special thanks to the ASPB and the ASPB Publications Committee for supporting this initiative. With these new members, we are pleased to note that the expanded board broadly reflects the topical distribution of research published in *Plant Physiology*.

## Welcome New *Plant Physiology* AFEs (2023–2024)

- Dechang Cao (Institute of Botany, Chinese Academy of Sciences, China)
- Jiawen Chen (John Innes Centre, UK)
- Joke De Jaeger-Braet (University of Hamburg, Germany)
- Manuel Gonzalez-Fuente (Ruhr-University of Bochum, Germany)
- Henning Kirst (University of Cantabria, Spain)
- Yee-Shan Ku (Chinese University of Hong Kong, China)
- Aida Maric (University of Freiburg, Germany)
- Hannah McMillan (Duke University, USA)
- Sebastian Moreno (Sainsbury Lab Cambridge University, UK)
- Dyoni Oliveira (Center for Plant Systems Biology of VIB Ghent, Belgium)
- Lara Pereira (University of Sheffield, UK)
- Moona Rahikainen (University of Helsinki, Finland)
- Janlo Robil (Ateneo de Manila University, Philippines)
- Alaeddine Safi (Center for Plant Systems Biology of VIB Ghent, Belgium)
- Maria-Angelica Sanclemente (Utrecht University, the Netherlands)
- Henryk Straube (University of Copenhagen, Denmark)
- Jiaqi Sun (Shandong University, China)
- Kyle Swentowsky (Cold Spring Harbor Lab, USA)
- Ryo Yokoyama (Max Planck Institute of Molecular Plant Physiology, Germany)

The new AFEs are joining those who started in January of 2022, listed below, who will stay on for one more year to continue writing News and Views, help with the transition, and mentor the new AFEs.

- Alexandra Burgess (University of Nottingham, UK)
- Amanda Cardoso (North Carolina State University, USA)
- Jian Chen (Jiangsu University, China)
- Gustaf E. Degen (University of Sheffield, UK)
- Marieke Dubois (Center for Plant Systems Biology of VIB Ghent, Belgium)
- Guadalupe Lucía Fernández-Milmanda (Center for Plant Systems Biology of VIB Ghent, Belgium)
- Yana Kazachova (Princeton University, USA)
- Amy Lanctot (Cold Spring Harbor Lab, USA)
- Divya Mishra (University of Wisconsin, USA)
- Míriam Osés-Ruiz (Universidad Publica de Navarra, Spain).
- Yadukrishnan Premachandran (Indian Institute of Science, Bangalore, India)
- José Manuel Ugalde (University of Bonn, Germany)
- Yajin Ye (Nanjing Forestry University, China)

